# The social fabric of the RNA degradosome^☆^

**DOI:** 10.1016/j.bbagrm.2013.02.011

**Published:** 2013-06

**Authors:** Katarzyna J. Bandyra, Marie Bouvier, Agamemnon J. Carpousis, Ben F. Luisi

**Affiliations:** a Department of Biochemistry, University of Cambridge, Tennis Court Road, Cambridge CB2 1GA, UK; b Laboratoire de Microbiologie et Génétique Moléculaires, CNRS et Université Paul Sabatier, 118 route de Narbonne, 31062 Toulouse, France

**Keywords:** RNA decay, RNA processing, Ribonuclease, RNase E, RNA degradosome, sRNA

## Abstract

Bacterial transcripts each have a characteristic half-life, suggesting that the processes of RNA degradation work in an active and selective manner. Moreover, the processes are well controlled, thereby ensuring that degradation is orderly and coordinated. Throughout much of the bacterial kingdom, RNA degradation processes originate through the actions of assemblies of key RNA enzymes, known as RNA degradosomes. Neither conserved in composition, nor unified by common evolutionary ancestry, RNA degradosomes nonetheless can be found in divergent bacterial lineages, implicating a common requirement for the co-localisation of RNA metabolic activities. We describe how the cooperation of components in the representative degradosome of *Escherichia coli* may enable controlled access to transcripts, so that they have defined and programmable lifetimes. We also discuss how this cooperation contributes to precursor processing and to the riboregulation of intricate post-transcriptional networks in the control of gene expression. The *E. coli* degradosome interacts with the cytoplasmic membrane, and we discuss how this interaction may spatially organise the assembly and contribute to subunit cooperation and substrate capture. This article is part of a Special Issue entitled: RNA Decay mechanisms.

## Highlights

• The organisation of the bacterial RNA degradosome
• The role in riboregulation and proposal for mechanism
• Discussion of access to substrates
• Discussion of the function of compartmentalisation

## 1 Introduction

**Fig. 1:**
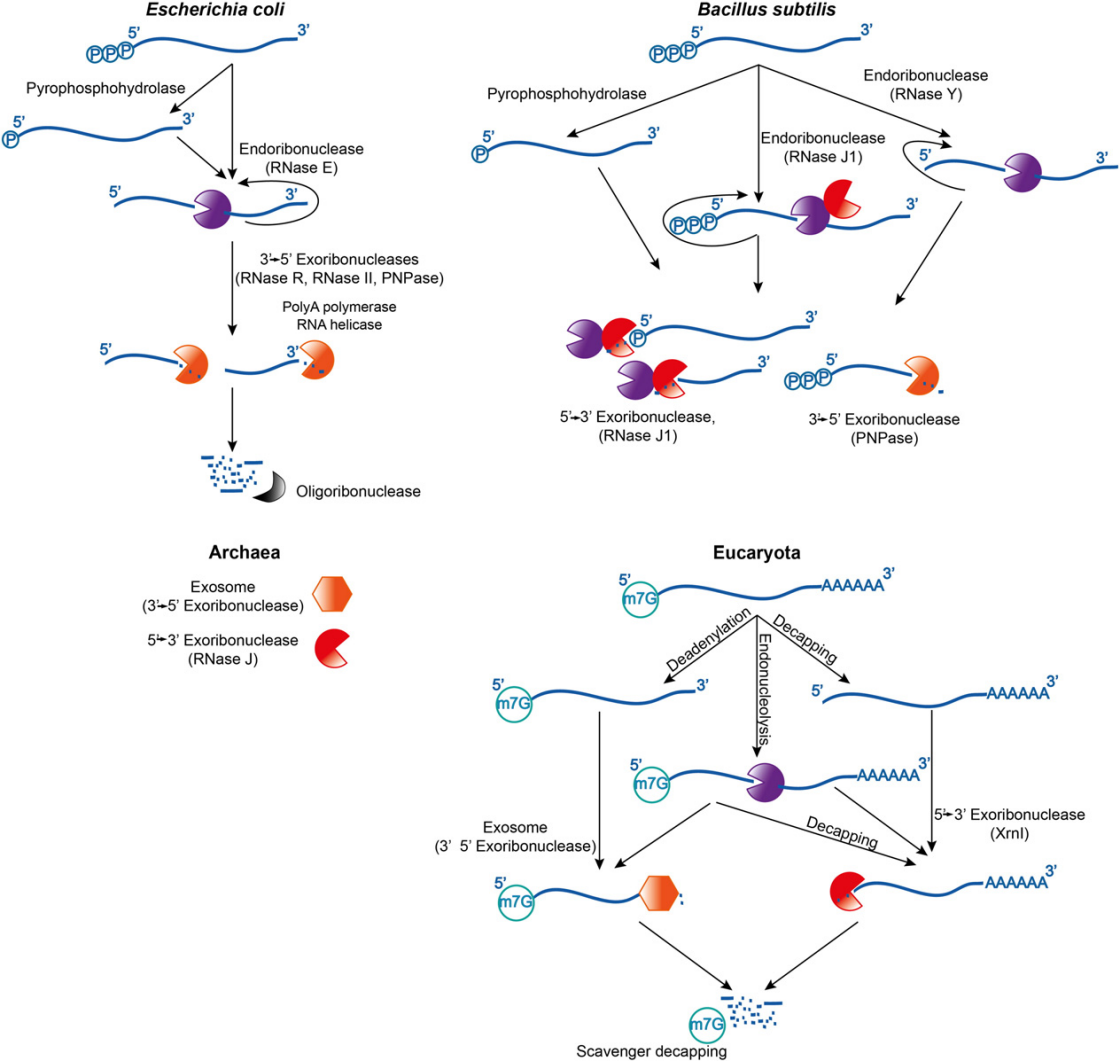
Overview of RNA decay pathways in the different life domains. Within the two main bacterial lineages, represented by the gram-negative *Escherichia coli* and the gram-positive *Bacillus subtilis* (top left and top right panels, respectively), the enzymes differ although the pathways share similarity. In both gram-negative and gram-positive lineages, the endoribonucleases can cleave the substrates repetitively, and the products are attacked by exoribonucleases. The degradation pathways in eukaryotes are shown for comparison.

Messenger RNAs are the short-lived information quanta of the hereditable genome. In all organisms, mRNA transcripts have limited but adjustable lifetimes [1–4], and their instability permits the expression of genetic information to be modulated in strength and duration so as to meet cellular requirements for homeostasis and timely response to environmental change. A key event in the degradation of a transcript in *Escherichia coli* and related γ-Proteobacteria is an initial cleavage by an endoribonuclease (Fig. 1). Once cleaved, transcripts are degraded to completion by exoribonucleases and oligoribonucleases through the cooperation with numerous enzymes, such as poly(A) polymerase and RNA helicases which facilitate the access to the RNA fragments. Fig. 1 summarises this decay pathway and for comparison shows representatives from all life domains. The pathways differ in detail among the three domains of life, and few of the components share common evolutionary origin; nonetheless, there are parallels in these pathways that perhaps reflect convergent evolutionary solutions to the common requirement for regulated transcript turnover.

In many bacteria, one of the key enzymes of the decay pathway is the hydrolytic endoribonuclease RNase E, highlighted in the *E. coli* decay pathway shown in the left panel of Fig. 1. In *E. coli* and other γ-Proteobacteria, RNase E is involved in the turnover of most mRNAs [5,6]. With the first cleavage by this enzyme, the transcript is destined to a fate of complete destruction. However, RNase E also takes part in RNA maturation processes and cleaves precursors of structured RNAs in a more controlled manner, so that the cleavage products are not marked for destruction but rather processed further to form a functional RNA molecule [5].

**Fig. 2:**
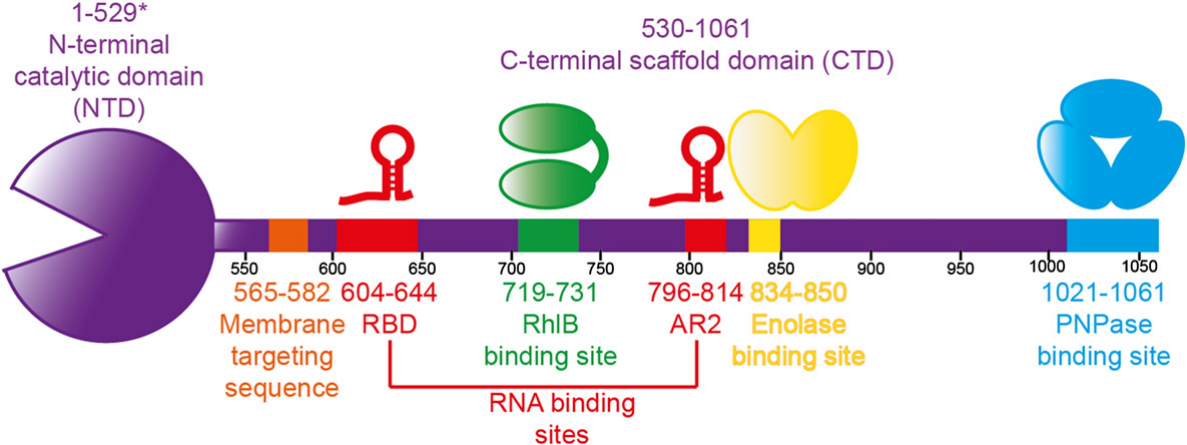
Components of the canonical degradosome. The asterisk marks the predicted size of the catalytic domain of RNase E; the amino acids 511–529 were not visible in the crystal structure [10].

One of the many fascinating facets of RNase E is its preference for substrates having a monophosphate group on the 5′ terminus [7]. As bacterial primary transcripts have a 5′ terminal triphosphate group, some are protected from cleavage until they are converted to a 5′ monophosphate form by pyrophosphohydrolase activity [8,9]. RNase E recognises the 5′ end of the RNA through contacts made by the catalytic domain, which is at the N-terminus of the protein and comprises roughly half its mass [10,11] (Fig. 2).

Although an accessible 5′ monophosphate can be a potent activator of RNase E, there are some RNA substrates for which the status of the 5′ end has little effect on the cleavage rate by RNase E, and for these the endoribonuclease probably cleaves by an internal entry without interacting with the 5′ end [12–16]. The mechanism for bypassing the 5′ end phosphate includes a supporting role by the non-catalytic C-terminal domain outside the N-terminal catalytic domain [12,14–19] (Fig. 2). The interplay of these two segments will be an important theme of this review.

In *E. coli* and many other bacteria, the non-catalytic portion of RNase E forms a scaffold for the physical association of other key enzymes of RNA metabolism into a multienzyme complex, known as the RNA degradosome [20–23]. Through this interaction, the enzymatic components can cooperate to turn over RNA [15,16,24,25]. The canonical components of the *E. coli* degradosome include RNase E, which recruits the DEAD-box RNA helicase RhlB [26,27], the glycolytic enzyme enolase [27–29], and the phosphorolytic exoribonuclease polynucleotide phosphorylase [30] (PNPase, Fig. 2). The degradosome scaffolding domain also includes RNA binding domains (RBD and AR2) [15,31], and a membrane targeting sequence [32] (MTS), which will be described in further detail in a subsection below. The scaffolding region is predominantly unstructured, but its interactions with other macromolecules are mediated through recognition modules that are structural microdomains [18,20].

RNase E occurs in many bacterial lineages, and is present in some bacteria that are distantly related to *E. coli*, such as *Streptomyces coelicolor* [33], as well as in some mycobacteria [34]. However, the components of the degradosome are not conserved, even among the γ-Proteobacteria [25,35–40]. The most common composition of proteins associated with RNase E includes a DEAD-box helicase, metabolic enzymes such as enolase or aconitase, and exoribonucleases such as PNPase or RNase R. Moving to very distantly related species, RNase E is entirely absent; a notable example is the model gram-positive bacterium *Bacillus subtilis* [41] which is divergent from *E. coli* by perhaps two billion years (Fig. 1). Although it lacks an RNase E homologue, *B. subtilis* and its relations use entirely different ribonucleases that fulfil the same function: RNase Y and RNase J [42–45]. RNase J is a member of the β-metallolactamase structural family, and it is important to note that this fold is entirely different from that of RNase E, implying that the two enzymes bear no common evolutionary ancestry. Multi-enzyme degradosome-like assemblies may also be present in *B. subtilis* [46,47], but this issue is under active debate [43,48]. A complex of RNase J with an RNA helicase has been identified recently in the pathogen *Helicobacter pylori* [49].

## 2 RNase E catalytic domain and its accommodating quaternary structure

**Fig. 3:**
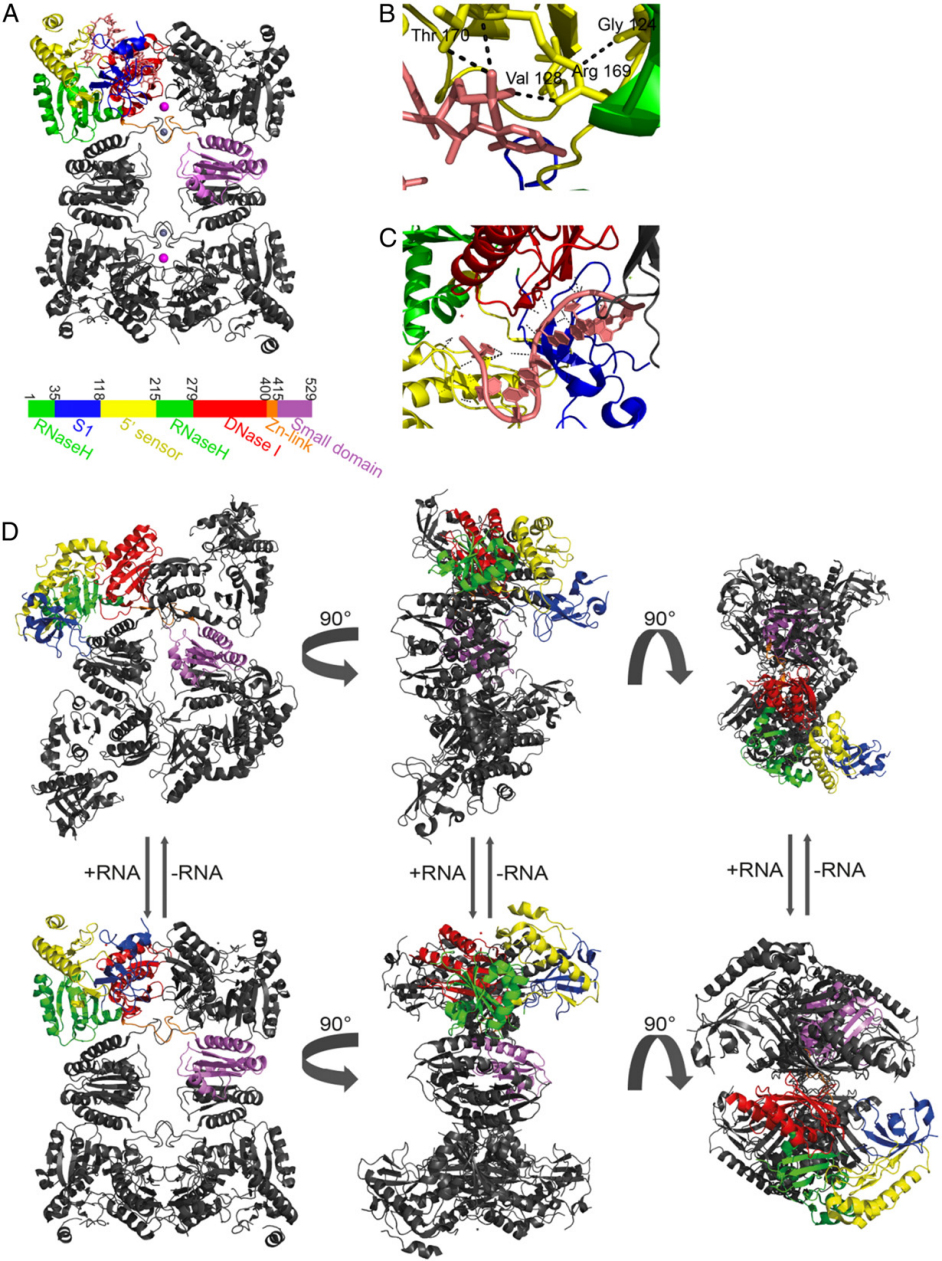
The quaternary structure of RNase E and its modes of structural change: A) Structural domains of RNase E (color coded) with a 13mer RNA oligo bound (showed only in one protomer for clarity) and the quaternary structural organisation. The tetramer is a dimer-of-dimers, and the principal dimer pairs are at the same horizontal level and are linked by a zinc ion (small grey sphere). B) The 5′ phosphate binding pocket of RNase E with the RNA bound; the main amino acids contacting the phosphate group are labelled. C) The RNA substrate tracking along a shallow groove at the active site, with the contacts between RNA and RNase E marked in black. D) Domain movement in the RNase E catalytic domain upon substrate binding, and quaternary structure flexibility. The left panel shows the overall structure of RNase E tetramer alone (top) and the RNA bound (bottom). The movements at the dimer-of-dimer interface are shown in the middle panel. Domain closure with the S1/5′-sensor domain clamping down on the substrate is demonstrated on the right panel.

Crystallographic studies of the catalytic domain of *E. coli* RNase E have provided insight into several of the fascinating features of RNase E activity (Fig. 3A). The catalytic domain forms a homotetramer that is organised as a dimer-of-dimers, in which the protomer pairs within each of the principal dimers are linked through organometallic bonds with a shared zinc ion [10,50]. The active site of the enzyme is formed between DNase I domains of two neighbouring protomers where coordinated magnesium ions present activated water for RNA cleavage.

Whilst RNase E has little apparent sequence specificity, it nonetheless prefers to cut RNA within single-stranded regions that are enriched in A/U [5]. The crystal structure shows that the single-stranded substrate tracks along a shallow groove at the active site, and this groove does not provide any base-specifying contacts; consequently, the apparent sequence preference for A/U is most likely due to preference for single stranded conformation [10] (Fig. 3C). Preferences for a purine at two bases to the 5′ side of the scissile phosphate [51] may be due to interactions of the purine ring with the S1 domain in the closed conformation.

The catalytically activating effect of having a 5′ monophosphate on certain substrates can be rationalised by the interaction of this chemical signal with a defined pocket in a domain referred to as the 5′ sensor domain (Fig. 3B). The 5′ end of the RNA fits into this pocket but must be single stranded to gain access. The interaction of the phosphate with the pocket is proposed to favour domain closure and boost catalytic activity, by increasing the catalytic rate and decreasing the K_m_ for the enzyme as seen in the RNase E paralogue, RNase G [11,52]. Marked structural changes also accompany substrate binding, with the S1/5′ sensor domain moving together as a unit to clamp down on the single stranded substrate in the active site [50]; Fig. 3C shows this structural transition of the S1/5′ sensor. The domain closure is proposed to orientate and present the phosphate backbone of the RNA for hydrolytic attack by water coordinated to the magnesium co-factor [10]. It might also help to co-recruit magnesium factor with the substrate, as occurs for instance in human DNA polymerase η [53].

The interfacial contacts between the domains act like ball bearings to accommodate changes in quaternary structure. In contrast, the principal dimer interfaces remain invariant with quaternary transitions (Fig. 3D). These quaternary structural changes could possibly occur when the ribonuclease interacts with complex RNA species, for instance during processing of precursors of folded RNA, or whilst maintaining a grip on the cleaved products during processive degradation of lengthy transcripts.

## 3 Two domains for RNase E, and two pathways for RNA decay

Deletion of RNase E is lethal in *E. coli* and other RNase-bearing bacteria [54], as is the mutation of the key catalytic residues [11]. Given the role of 5′ sensing in stimulating RNase E activity, and the potential to boost sRNA action [55], it might seem surprising that inactivating point mutations in the pocket are not lethal [11,19]. These findings suggest that the pathway of 5′ end sensing is neither the sole, nor the most important pathway for RNase E mediated RNA turnover. This is in accord with the viability of a deletion mutant for the pyrophosphohydrolase RppH [9], proposed to decap transcripts to leave a 5′ monophosphate terminus that would activate RNase E cleavage. The absence of RppH in the *E. coli* cells was found to significantly impair pyrophosphate removal from the rpsT P1 transcript resulting in a 3- to 5-fold increase in stability of this mRNA; globally, the lack of RppH influences the half-life of about 400 protein coding transcripts [9]. However, RppH is one of more than a dozen known Nudix superfamily pyrophosphohydrolases in *E. coli*, and potentially they can act redundantly [56].

**Fig. 4:**
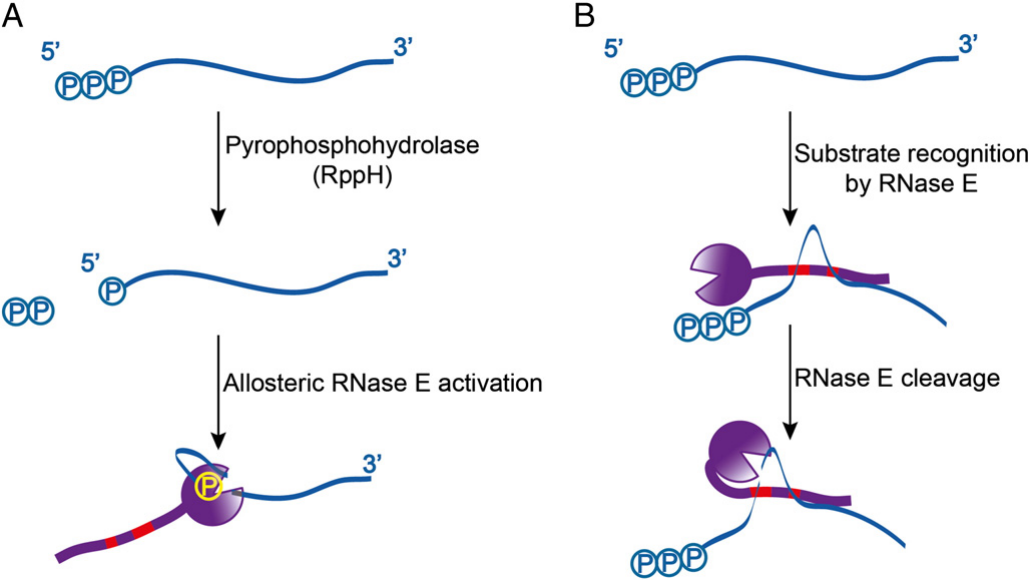
A cartoon schematic of two pathways for RNA cleavage by RNase E and the degradosome: 5′ end sensing and the 5′ bypass pathway. A). Activation of RNase E by 5′ end sensing to trigger domain closure. B). Proposed model for how the CTD might affect 5′ end bypass by capturing and presenting potential substrates to the catalytic domain. The red bars represent the two RNA binding domains in the C-terminal half of RNase E.

Perhaps equally surprising to the non-essentiality of the 5′ end sensing is the viability of strains bearing deletion of the C-terminal domain, *i.e.*, the degradosome scaffolding domain [15,24,57]. Again, this suggests that the RNase E catalytic domain can mediate a degradation pathway that does not require the degradosome assembly. However, when combined with the 5′ sensing mutation, RNase E truncations are synthetically lethal [19]. Similarly, combining the RppH mutation and the RNase E truncation is also synthetically lethal [17]. These observations indicate that there are two, parallel pathways for RNA degradation through RNase E: one requiring the 5′ end monophosphate and one depending on the RNA fold (Fig. 4) [18].

Mutations affecting Rho-dependent transcription termination can overcome synthetic lethality by a pathway that requires RNase H. It has been proposed that RNase H cleavage of RNA–DNA hybrid ‘R-loops’ formed during transcription might substitute for RNase E-dependent RNA processing and mRNA degradation [17].

As described above, the 5′ end pathway involves domain closure triggered when the free 5′-monophosphate can become engaged in the sensing pocket. For the mechanism involving 5′ end bypass, we envisage that the single stranded region, perhaps as little as 5 bases in length, will still form the substrate: either the S1 domain is engaged to present the phosphate backbone to the active site, or the enzyme is in an open conformation, with the fold of the RNA facilitating substrate presentation. This mode of interaction does not necessarily require interaction with the 5′ terminus of the RNA. We also propose that the C-terminal domain of RNase E and its degradosome partners help in presenting the substrate to the catalytic domain. The interactions could be mediated by the RBD and AR2 [15,58], and possibly by the basic C-terminal tail of RhlB [26], and may be favoured with the interaction with folded RNA species. PNPase could also assist through its KH and S1 RNA-binding domains [59–61]. There is likely to be interplay between the 5′ end-dependent and independent pathways and they may not be exclusive. For instance, there could be cooperation of the RNA binding domains in the C-terminal half of RNase E to present structured substrates to the catalytic domain which would recognise the 5′ end and cleave them more efficiently, and to help recruit and present small non-coding RNAs (sRNAs) or sRNA/Hfq complexes for pairing to the mRNA target [55]. We turn now to explore this topic.

## 4 RNase E, the degradosome and sRNA mediated riboregulation

Riboregulation in bacteria is an important aspect of the control of gene expression, especially in response to stress [62,63]. This regulatory mode is mediated by small non-coding RNAs of 50 to 300 nucleotides that share imperfect sequence complementarity with the target RNAs. Despite the limited size of the pairing region, the sRNAs recognise targets with selectivity and affect rapid responses, which is most often by repressing translation and triggering degradation of the targeted mRNA. Many of the sRNAs studied in *E. coli* and *Salmonella sp.* act in conjunction with the RNA chaperone Hfq, which assists the sRNAs to pair with their target mRNAs and also protects them from premature degradation [64]. The main body of the sRNA might interact with Hfq such that the “seed region” is presented to the transcript for cognate base-pairing [65–67]. The primary nuclease through which sRNAs trigger transcript instability is RNase E [68,69], and once RNase E initiates cleavage, degradation proceeds rapidly for both mRNA and sRNA, so that their turnover is effectively coupled [70].

Truncations of RNase E that lose the degradosome scaffolding domain are deficient for some small RNA mediated responses. Deletion of this portion of *Salmonella* RNase E (residues 702 to 1061) weakens the sRNA-mediated repression of the outer membrane protein OmpD [71]. Similar deletions decrease the degradation rate of the sRNAs [72], and diminish the effectiveness for target gene silencing [70,73]. Some portion of the C-terminal domain may be important for mediating the repression effects of sRNAs, and one model proposes that these domains may help to recruit the Hfq:sRNA complex [74,75]. Binding data suggest that RNA can bridge between Hfq and the RNA-binding domains that are located in the C-terminal half of RNase E [76]. In this sense, the large degradosome mediated sRNA/Hfq “recognition assembly” has some parallels to the Dicer machinery of eukaryotes. The interaction of these RNA-binding domains of RNase E may help to present the seed region of sRNA, and also assist the delivery of the target to the catalytic domain of RNase E for cleavage. We envisage a mechanism for this action in which an sRNA activates the catalytic domain of RNase E, whilst other components of the degradosome assembly together with RNA binding domains in the C-terminal half of RNase E might interact with the mRNA target to aid presentation of the target site to the catalytic groove of RNase E [55].

## 5 How riboregulation might enable substrate access on polysomes

Prévost et al. [73] have reported a case in which an sRNA can direct RNase E to cleave targets at a distance from the seed-pairing region. The sRNA RyhB base pairs with the target *sodB* mRNA (encoding superoxide dismutase) at the 5′-UTR (untranslated region), and this induces RNase E to cleave within the coding region during active translation. Like many other cases of sRNA mediated riboregulation, the mechanism of RyhB involves the RNA chaperone, Hfq. Binding of RyhB at the 5′-UTR triggers two processes in sequence: first, prevention of a new round of translation, by simply occluding the ribosome binding sites; second, RNase E cleavage at the distal site after it has been cleared of translating ribosomes. This two-stage mechanism avoids the fate of accumulating ribosomes that are trapped on a cleaved, stop-less transcript [73].

**Fig. 5:**
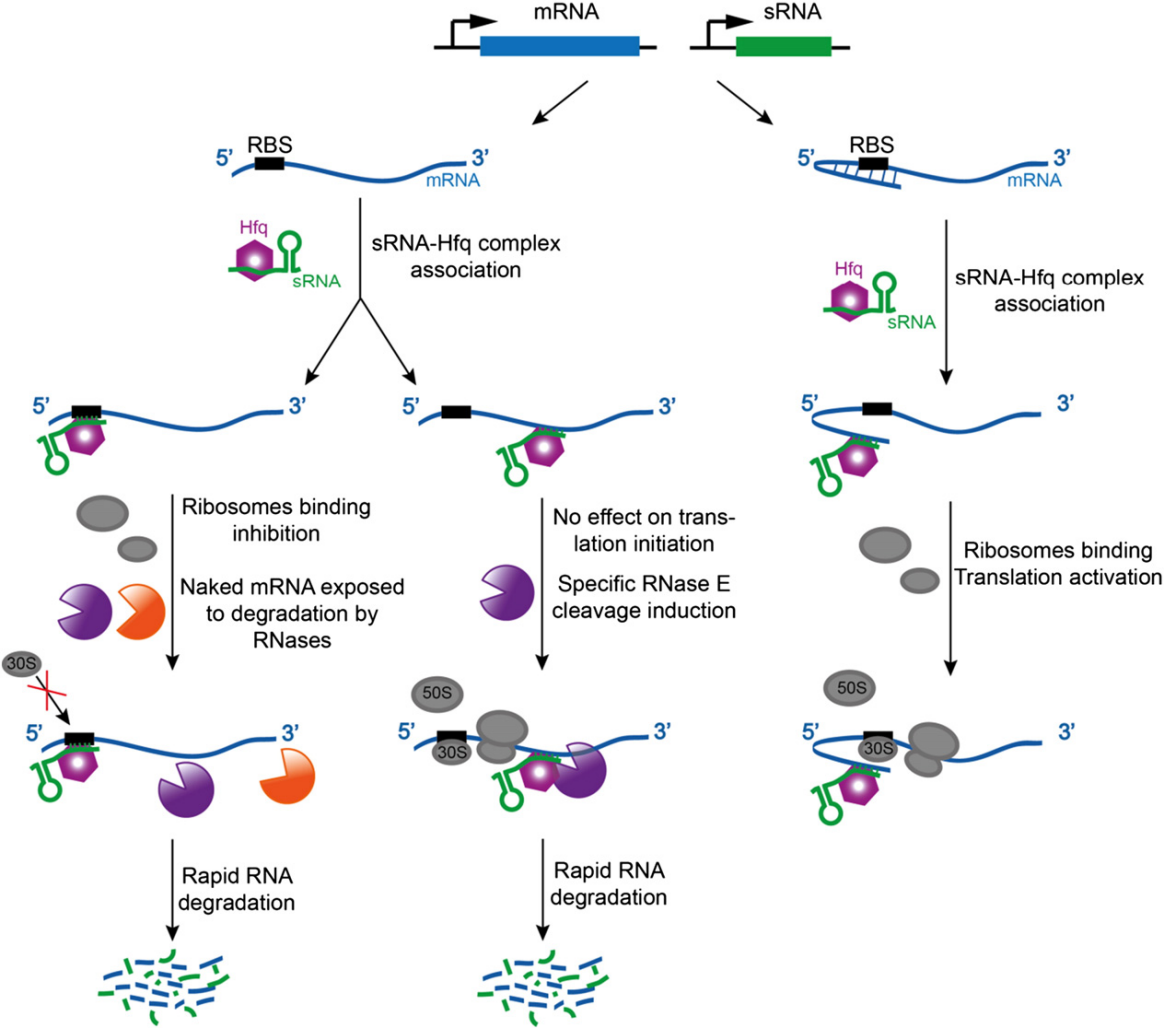
RNase E, the degradosome and sRNA mediated post-transcriptional regulation. Cartoon schematic of the sRNA mediated pathways for degradation (left and central panels) and translational activation (right panel). The purple body represents RNase E and the orange body is an exoribonuclease.

One model that may account for sRNA mediated action, both locally and at a distance, is based on evidence that the degradosome may bind to polysomes in *E. coli* [77]. The interaction is mediated by the AR2, RBD, and the highly basic C-terminal tail of the RhlB helicase. Interacting with polysomes, the degradosome might remain associated with the sRNA/Hfq/mRNA region whilst the emerging transcript spools from the terminal ribosome. This could continue until a structural signal is recognised by a component of the degradosome, and this would trigger cleavage of the exposed transcript. Although such a process enables the graceful exit from translation without generating stop-less codons, it is conceivable that cleavages might be made within the coding region of an actively translated transcript; this would result in an “emergency stop” that would generate a stop-less mRNA and would require rescue of the stalled ribosome for example by the tmRNA system. For instance, this could enable rapid termination of biosynthesis of proteins that could cause cell death in stress conditions, like porin synthesis during envelope stress response (Fig. 5B).

Translating ribosomes are potent helicases [78], and it might be expected intuitively that it would be difficult for an sRNA or even the large degradosome mediated sRNA/Hfq “recognition assembly” to transiently stall such a machine. However, there may be small windows of opportunity kinetically that enable a site to be rapidly exposed and cleaved, and this would ensure that subsequent rounds of translation are prevented. Such pausing could be triggered by ribosomes encountering certain signals in the message or regulatory signals (such as hibernation factor). This hypothetical mode would require collaboration with the tmRNA pathway to rescue the terminated assembly. Similar or analogous effects of ribonucleases may occur in other bacteria; for instance, an interaction of a ribonuclease (RNase J) and ribosomes is observed in *H. pylori* [49]. This process is not expected to occur under exponential conditions, but might occur with potentially deleterious stress or other conditions that require rapid adaptation.

## 6 Cell localisation and compartmentalisation

The subcellular compartmentalisation of enzymes is likely to influence strongly access to substrates. In *E. coli*, RNase E is localised to the cytoplasmic membrane [79–81]. The localisation can be visualised *in situ* by fluorescence microscopy of living cells expressing fusions of RNase E with fluorescent proteins that are encoded either on plasmids or the chromosome, and the protein moves on the membrane surface in a highly dynamic manner (AJC and BFL, unpublished). The membrane localisation of RNase E is mediated principally by an amphipathic α-helix localised 30 to 45 residues to the c-terminus of the catalytic domain (residues 565–582 in *E. coli* RNase E), which is conserved in RNase E homologues of the β- and γ-Proteobacteria [79]. Similar ‘membrane anchors’ are found in other membrane-associated proteins in bacteria, such as the actin-like MreB, the cell division proteins FtsA and MinD, and the signal recognition particle receptor FtsY [82–85].

The membrane anchor is envisaged to float on the surface of the inner leaflet of the cytoplasmic membrane, such that the hydrophobic residues are immersed in the hydrocarbon interior of the lipid membrane and the basic residues form electrostatic interactions with the polar head groups [79]. This model is consistent with the results of molecular dynamics simulations of peptide binding to vesicles having the complex lipid composition of *E. coli* membranes (Syma Khalid, personal communication). The RNase E catalytic domain by itself may associate with the cytoplasmic membrane, and this affects ribonuclease activity by stabilising the catalytic core [80] although this effect is likely due to a general electrostatic attraction.

The compartmentalisation of RNase E and the degradosome may be a specialisation of the β- and γ-Proteobacteria, as RNase E of other species generally lacks identifiable membrane anchors. In the α-proteobacterium *Caulobacter crescentus*, RNase E is not on the membrane and appears to be associated with the nucleoid [86]. However, in the highly divergent *B. subtilis*, the degradosome component RNase Y is membrane-associated [87]. In those cases where the ribonuclease is membrane-localised, the role of the membrane interaction has not been established unequivocally, but it is clear that the interaction is required for competitive fitness since disruption of the membrane anchor causes slow growth in *E. coli* [79]. The interaction of *B. subtilis* RNase Y with the membrane may be required for its activity *in vivo* [87].

Cryo-electron microscopy of the wild type degradosome suggests that the sample may be conformationally heterogeneous (Jarrod Voss, personal communication), but it is likely that membrane association may influence and help to confine the spatial spread of the components. We speculate that this membrane association structurally organises the RNase E tetramer, as it would bring the non-catalytic C-terminal domains to the periphery of a focal point of catalytic domains (Fig. 7).

## 7 Summary and perspective

A transcript's lifetime will be affected by its accessibility, and accordingly, active translation that enshrouds the transcript with ribosomes protects mRNAs [15,88,89]. The available data indicate that each transcript has a characteristic and tunable lifetime, and properties of the RNA such as propensity to fold as it issues from the last bound ribosome (or polymerase itself), will affect the rate at which it can be attacked by a ribonuclease. In a T7 RNA polymerase-based system, the 8-fold higher transcription rate uncouples transcription from translating ribosomes, which are too slow to keep pace with the polymerase. The resulting ribosome-free mRNAs are extremely sensitive to ultra-rapid degradation that is mediated by RNase E [16].

**Fig. 6:**
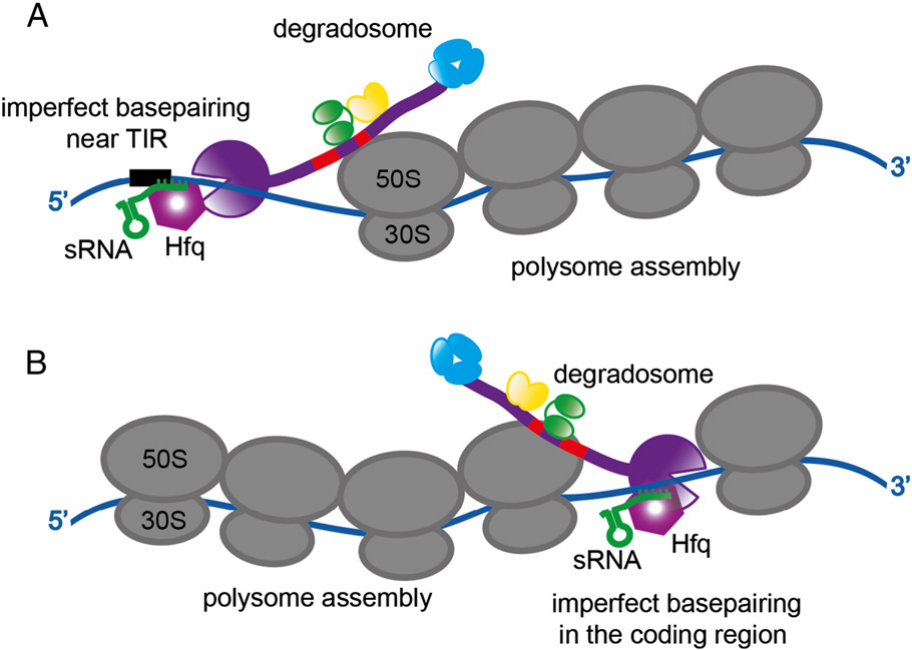
A speculative model for interaction of the degradosome and polysome in sRNA mediated gene silencing [77]. Panel A suggests a mechanism for sRNA mediated activation of RNase E on the 5′ end of a spooling transcript. B) A more radical “emergency stop” process to terminate translation prematurely. Here, the cleavage is mediated in the coding region of an actively translated mRNA. This hypothetical mode would generate a stop-less transcript and require collaboration with the tmRNA pathway to rescue the terminated assembly. RNase E is purple, and the two RNA binding sites in the C-terminal half of the enzyme are shown in red. Helicase is the bi-lobed green body, enolase is the yellow dimer and PNPase is the blue trimer.

Under normal conditions in *E. coli* where transcription is driven by *E. coli* RNA polymerase, it seems likely that the decay is initiated at some stage during translation — and this could be either as the message exits from the trailing ribosome (Fig. 6A), or perhaps more radically, internally in a translating polysome (Fig. 6B for example). Once the attack is initiated, it is critical that degradation continues to completion, and speculatively the degradation machinery might be waiting in a passive mode on polysomes for stochastic access to the issuing end of the transcript or for activating signals from sRNAs or pyrophosphohydrolases, that can help to recruit the substrate and allosterically activate the catalytic domain of RNase E for cleavage under stress or rapidly changing conditions [77]. A recent report suggests that the density of translating ribosomes is an important factor in RNA degradation by RNase E, where 5′ monophosphate generated by the pyrophosphohydrolase RppH accelerates the cleavage by RNase E in 5′ UTR of the transcripts with poor ribosome binding affinity [89]. In considering such a dynamic picture, it must also be borne in mind that the degradosome might be generally busy with processing tRNA and rRNA, thereby limiting its availability for mRNA decay [90]; perhaps such a distracting effect may potentially link degradative activity with cellular growth conditions.

**Fig. 7:**
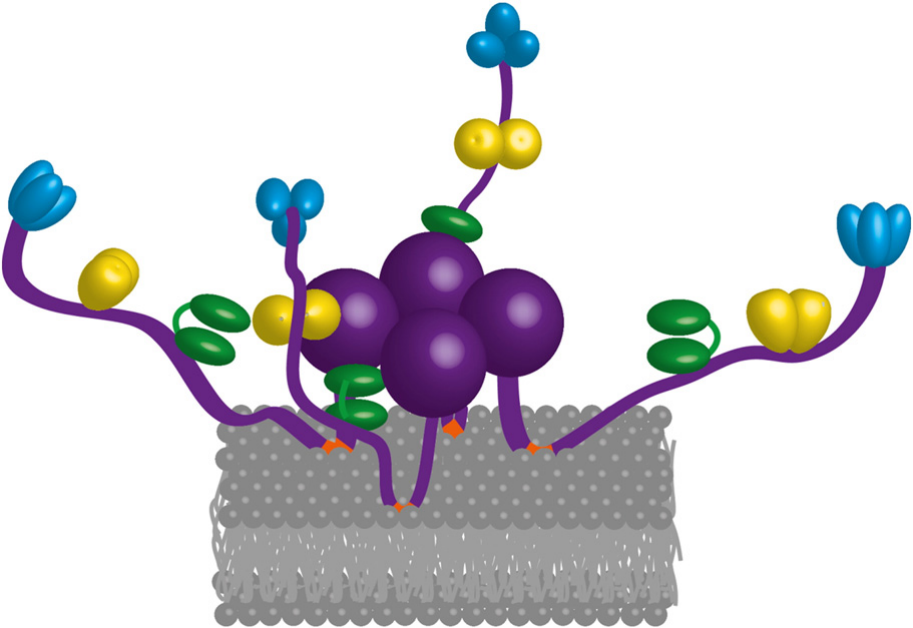
The membrane localisation of *Escherichia coli* RNase E and the RNA degradosome. Schematic of proposed association of an RNase E tetramer (purple) on the inner leaflet of the cytoplasmic membrane through four amphipathic alpha helices (orange). With this model, the hydrophobic side chains of the amphipathic helices are partially immersed in the hydrocarbon interior of the lipid bilayer (gray lines). The grey spheres are the lipid polar head groups. Membrane association may help to bring the other degradosome components closer in space. Each purple sphere is an RNase E catalytic domain, RhlB helicase is the bi-lobed green body, dimeric enolase is shown in yellow and trimeric PNPase in blue.

The cooperation of components of the degradosome facilitates substrate turnover, and the assembly is likely to be highly flexible and accommodating for complex RNA folds, with interesting functional consequences for substrate capture and subunit communication. Some of the structural subdomains forming the N-terminal half of RNase E can move relative to each other with substrate binding (*e.g.*, the S1-5′ sensor domain) and quaternary structure of the tetramer allows changes at the dimer-of-dimer interfaces (Fig. 3). The scaffolding region of the degradosome, namely the C-terminal region of RNase E, is also highly flexible, and this would help to confer interactions and cooperation between degradosome components. Tethering of the degradosome to the membrane would help to bring the components into juxtaposition, with capacity for flexible maneuvering. Thus, the entire assembly on the membrane might resemble something like a sea anemone, with flexible tentacles that can capture and engulf substrates (Fig. 7). Although the C-terminal domain of RNase E is predicted to be predominantly unstructured, it is not expected to be elongated and in fact the degradosome might nonetheless be a semi-compacted assembly. The available EM data suggest that the particles might be 30 nm in diameter, which suggests that there is compaction that would limit the ‘throw’ of the segments into the cellular milieu (Górna PhD thesis). We envisage that the flexibility may enable the degradosome assembly to scan along translating polysomes, and facilitate the identification of suitable substrates to initiate degradation. These hypotheses, as well as further insight into the organisation and activities of the degradosome, require challenging experimental approaches to explore the degradosome structure on the cytoplasmic membrane in a living cell.
